# Surgical treatment for thyroid carcinoma: retrospective study with 811 patients in a Brazilian tertiary hospital

**DOI:** 10.1590/2359-3997000000209

**Published:** 2016-09-26

**Authors:** Beatriz G. Cavalheiro, Leandro L. Matos, Ana Kober N. Leite, Marco Aurélio V. Kulcsar, Claudio R. Cernea, Lenine G. Brandão

**Affiliations:** 1 Departamento de Cirurgia de Cabeça e Pescoço Faculdade de Medicina da Universidade de São Paulo Instituto do Câncer do Estado de São Paulo São Paulo SP Brasil Departamento de Cirurgia de Cabeça e Pescoço, Faculdade de Medicina da Universidade de São Paulo (FMUSP) e Instituto do Câncer do Estado de São Paulo (ICESP), São Paulo, SP, Brasil

**Keywords:** Thyroid diseases, thyroid neoplasms, epidemiology, prognosis

## Abstract

**Objective:**

The aim of the present study was to describe the epidemiologic data, histological type, treatment and follow-up of the 811 patients treated for thyroid cancer in Instituto do Câncer do Estado de São Paulo (ICESP) over 5 years.

**Materials and methods:**

Retrospective analyses of electronic chart information.

**Results:**

There were 679 cases (83.7%) of papillary thyroid cancer, 61 (7.5%) of follicular carcinoma, 54 (6.7%) of medullary carcinoma, 11 (1.4%) of poorly differentiated carcinoma and 6 of anaplastic carcinoma (0.7%). The majority of patients were female (82.2%), and the mean age was 50.5 ± 15 years. Two hundred forty-two patients had disease persistence or recurrence. At the last follow-up, 629 (77.6%) patients were alive and disease free, 141 (17.4%) were alive with disease, and 41 (5.1%) were deceased, with 37 deaths related to thyroid cancer.

**Conclusion:**

This study was able to outline the profile, disease type and evolution of patients treated for thyroid cancer at a single tertiary hospital.

## INTRODUCTION

Thyroid cancer (TC) is not one of the most prevalent cancers in the population, but its incidence has been increasing continuously, primarily because of papillary thyroid cancer (PTC). The increase in TC diagnosis is explained in part by the improvement of diagnostic techniques, the wide use of ultrasound and fine-needle aspiration cytology and better pathological analyses. Although the increase has primarily been of small lesions, there has also been an increase in nodules over 2 cm, which shows us that there has been a real increase in TC incidence and not just better screening (1).

The estimated risk for thyroid cancer in the Brazilian population in 2014 was 1.15 cases per 100,000 men and 7.91 cases per 100,000 women. In São Paulo, the incidence of new cases per year was 1,000 patients, and 15,955 new cases were diagnosed from 1997-2008. From 1997-2000 and from 2005-2008, the incidence increased 5.2% per year and was highest among females below age 50 years. PTC was the main histologic type related to the incidence increase, and there was a decrease in other histological types (2).

Disease progression and mortality are extremely variable in TC, depending on the histologic type. Differentiated thyroid cancer (DTC; follicular and papillary types) is usually an indolent disease that with adequate treatment has a very good prognosis. The literature reports that less than 5% of patients die from the disease within 10 years. However, poorly differentiated and anaplastic carcinomas have a very poor prognosis, with a mean survival of less than 6 months in the anaplastic cases (1,2).

The aim of this study was to describe the epidemiology, pathology characteristics, treatment and follow-up of patients treated for TC at a single tertiary hospital in São Paulo, Brazil.

## MATERIALS AND METHODS

The charts of the patients treated for TC in *Instituto do Câncer do Estado de São Paulo* (ICESP) from May 2009 to January 2014 were reviewed. The study was approved by the IRB under the number 228/14.

ICESP is a tertiary oncologic hospital that opened in 2009. It is maintained by the government of the State of São Paulo, and it is a hospital linked to the University of São Paulo Medical School. The Head and Neck Surgery Department began its activities in the institution in May 2009 and since then has been progressively increasing the number of clinic appointments, surgeries and medical staff (attending and residents).

Between May 2009 and January 2014, 811 patients were surgically treated for TC in ICESP. Patients included those who were receiving initial treatment and those who had recurrent/persistent disease and were referred from another institution.

In cases of well and moderately differentiated thyroid carcinomas, the surgical procedure of choice was total thyroidectomy. Neck dissection was only performed in cases of confirmed cervical metastases (clinical or radiological) in patients with well-differentiated carcinomas, and was performed electively in patients with medullary carcinoma (central neck compartment). There were some cases in which adjacent lymph nodes were resected with the thyroid and that gave us an “N” stage even if a formal central neck dissection was not performed. While surgical procedures are well established for well-differentiated and moderately differentiated thyroid carcinomas, it is not so with poorly differentiated or undifferentiated ones. Most of these cases had their histological diagnosis confirmed after total thyroidectomy, or in an attempt to do so. In such cases, we opted for the resection of as much tumor as possible prior to the indication of complementary therapies.

The variables sex, age at diagnosis, weight and height, lab tests related to thyroid metabolism, surgical procedure, pathologic findings – TNM system (3), other treatments and follow-up were analyzed.

Although it is an institutional policy always to analyze pathological specimens from other institutions, there was some data loss related to the pathological findings of previous surgeries, previous radioiodine treatment and dosage and other chart information that was not available. Moreover, because the hospital was founded in 2009, we do not have long follow-up period with our patients.

Excluded from the study were patients who were treated with nonsurgical options and patients with thyroidectomies performed for suspicious nodules that did not confirm malignancy.

During follow-up, patients had medical appointments with the endocrinology and head and neck surgery teams at maximal intervals of 6 months. Patients with differentiated thyroid cancer were submitted to TSH suppression based on the risk stratification and TNM classification.

Data were gathered and analyzed using Microsoft Excel^®^ software (Microsoft Corporation^®^, Redmond, WA, USA). Graphs and Kaplan-Meier curves were performed using SPSS^®^ 17.0 software (SPSS^®^ Inc; Ilinois, USA).

## RESULTS

Among the 811 patients surgically treated for TC, 667 (82.2%) were female, and 144 were male. The age of diagnosis for the women varied from 8-87 years, with a mean of 50 ± 15 years, and the body mass index (BMI) varied from 16.3-57.0, with a mean of 28.7 kg/m^2^. The age of diagnosis for male patients varied from 16-84 years, with a mean of 52.4 ± 15.6 years. The mean BMI was 27.2 kg/m^2^ (16.5-46.8 kg/m^2^).

One hundred eighty-two patients (22.4%) received initial treatment elsewhere and were referred to ICESP due to persistence or recurrence.

The majority of patients were submitted to total thyroidectomy (552), 110 were submitted to total thyroidectomy and central neck dissection, and 122 were submitted to total thyroidectomy with central and lateral neck dissection. Twenty-seven cases were considered inoperable.

As for the histological type, the absolute majority was PTC (679 cases, representing 83.7%), followed by follicular carcinoma in 61 patients (7.5%), medullary carcinoma in 54 patients (6.7%), poorly differentiated thyroid cancer in 11 patients (1.4%) and anaplastic carcinoma in six patients (0.7%).

**Figure 1:**
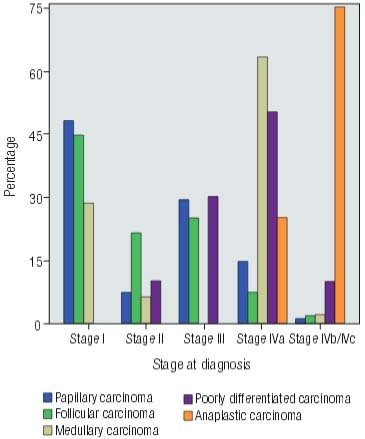
Bar graph demonstrating the frequency of each histological type of thyroid carcinoma according to the stage. The rates for papillary, follicular, medullary, poorly differentiated and anaplastic carcinoma were, respectively: 48.0%, 44.6%, 28.6%, 0.0% and 0.0% for stage I; 7.3%, 21.4%, 6.1%, 10.0% and 0.0% for stage II; 29.2%, 25.0%, 0.0%, 30.0% and 0.0% for stage III; 14.4%, 7.1%, 63.3%, 50.0% and 25.0% for stage IVa; and 1.1%, 1.8%, 2.0%, 10.0% and 75.0% for stage IVb/IVc.

Forty-three patients could not be correctly staged in the TNM system but it was noted a predominance of early stage disease (stages I and II) for DTC patients and a higher incidence of advanced disease (stage IV) for individuals with poorly differentiated and anaplastic carcinoma (Figure 1). Table 1 shows pathological staging related to sex, age, pathological findings and disease progression for TC patients. It also presents data related to recurrence, distant metastasis and status at the last follow-up.

**Table 1:** Descriptive data of patients included in the study stratified by histological type of primary tumor

**p Stage**	**N (%)**	**Age range (years)**	**Mean age (year)**	**Female sex**	**Mean diameter (cm)**	**Iodotherapy**	**Cervical recurrence**	**Distant metastasis**	**Disease persistence**	**Alive (disease free)**	**Alive (with disease)**	**Death**
**Papillary carcinoma (N = 679)**												

pT1a N0	205 (30.2%)	20-82	52.7	184 (89.8%)	0.5	28 (13.66%)	7 (3.4%)	0 (0.0%)	0 (0.0%)	203 (99.0%)	2 (0.8%)	0 (0.0%)
pT1b N0	88 (13.0%)	20-78	49.6	75 (85.2%)	1.4	57 (64.77%)	10 (11.4%)	3 (3.4%)	0 (0.0%)	83 (94.3%)	3 (3.4%)	2 (2.27%)
pT1a N1a	8 (1.2%)	21-48	34.6	6 (75.0%)	0.5	5 (62.5%)	0 (0.0%)	0 (0.0%)	0 (0.0%)	8 (100.0%)	0 (0.0%)	0 (0.0%)
pT1a N1b	6 (0.9%)	26-68	47.0	5 (83.3%)	0.8	6 (100.0%)	2 (33.3%)	0 (0.0%)	0 (0.0%)	6 (100.0%)	0 (0.0%)	0 (0.0%)
pT1b N1a	10 (1.5%)	20-71	42.5	8 (80.0%)	1.5	9 (90.0%)	1 (10.0%)	0 (0.0%)	0 (0.0%)	10 (100.0%)	0 (0.0%)	0 (0.0%)
pT1b N1b	9 (1.3%)	21-50	37.3	6 (66.7%)	1.5	9 (100.0%)	0 (0.0%)	1 (11.1%)	0 (0.0%)	8 (88.9%)	0 (0.0%)	1 (11.11%)
pT2 N0	48 (7.1%)	22-84	48.8	42 (87.5%)	2.8	40 (83.33%)	4 (8.3%)	1 (2.1%)	0 (0.0%)	47 (97.9%)	1 (2.1%)	0 (0.0%)
pT2 N1a	7 (1.0%)	14-87	39.7	4 (57.1%)	2.3	7 (100.0%)	3 (42.9%)	0 (0.0%)	0 (0.0%)	5 (71.4%)	2 (28.6%)	0 (0.0%)
pT2 N1b	5 (0.7%)	22-72	50.2	3 (60.0%)	2.5	4 (57.14%)	1 (20.0%)	1 (20.0%)	0 (0.0%)	5 (100.0%)	0 (0.0%)	0 (0.0%)
pT3 N0	141 (20.8%)	18-87	51.9	121 (85.8%)	2.3	122 (86.52%)	10 (7.1%)	6 (4.3%)	0 (0.0%)	132 (93.6%)	7 (5.0%)	1 (0.71%)
pT3 N1a	37 (5.4%)	19-80	46.8	34 (91.9%)	2.3	37 (100.0%)	12 (32.4%)	4 (10.8%)	0 (0.0%)	30 (81.1%)	4 (10.8%)	3 (8.11%)
pT3 N1b	39 (5.7%)	20-73	46.4	33 (84.6%)	3.0	36 (92.30%)	11 (28.2%)	5 (12.8%)	1 (2.6%)	27 (69.2%)	11 (28.2%)	1 (2.57%)
pT4aN0	7 (1.0%)	22-81	50.3	4 (57.1%)	6.7	7 (100.0%)	1 (14.3%)	3 (42.9%)	0 (0.0%)	6 (85.7%)	1 (14.3%)	0 (0.0%)
pT4a N1a	8 (1.2%)	44-65	58.0	5 (62.5%)	5.0	8 (100.0%)	1 (12.5%)	3 (37.5%)	3 (37.5%)	3 (37.5%)	4 (50.0%)	1 (12.5%)
pT4a N1b	14 (2.1%)	13-69	42.3	8 (57.1%)	4.5	12 (85.71%)	9 (64.3%)	4 (28.6%)	3 (21.4%)	6 (42.9%)	6 (42.9%)	2 (14.28%)
pT4b N0	3 (0.4%)	43, 54, 73	56.7	2 (66.7%)	9.8	2 (66.67%)	3 (100.0%)	2 (66.7%)	3 (100.0%)	0 (0.0%)	2 (66.7%)	1 (33.33%)
pT4b N1a	2 (0.3%)	4, 64	52.5	1 (50.0%)	8.0	0 (0.0%)	0 (0.0%)	1 (50.0%)	2 (100.0%)	0 (0.0%)	1 (50.0%)	1 (50.0%)
pT4b N1b	2 (0.3%)	50, 70	60.0	0 (0.0%)	5.0	1 (50.0%)	0 (0.0%)	2 (100.0%)	2 (100.0%)	0 (0.0%)	1 (50.0%)	1 (50.0%)
Missing stage	40 (5.9%)	19-78	47.7	31 (77.5%)	-	27 (67.50%)	12 (30.0%)	14 (2.1%)	5 (12.5%)	15 (37.5%)	22 (55.0%)	3 (7.50%)
Total	679 (83.72%)	20-84	48.2	572 (84.24%)	3.4	419 (61.71%)	87 (12.81%)	50 (7.36%)	19 (2.80%)	594 (87.48%)	67 (9.87%)	16 (2.36%)

**Follicular carcinoma (N = 61)**												

pT1 N0	10 (16.4%)	31-82	58.0	9 (90.0%)	1.4	5 (50.0%)	1 (10.0%)	3 (30.0%)	0 (0.0%)	7 (70%)	2 (20%)	1 (10%)
pT1 N1a	1 (1.6%)	38	38.0	1 (100.0%)	1.5	1 (100.0%)	1 (100.0%)	1 (100.0%)	0 (0.0%)	0 (0.0%)	1 (100.0%)	0 (0.0%)
pT2 N0	13 (21.3%)	19-75	48.8	11 (84.6%)	2.5	10 (76.9%)	0 (0.0%)	2 (15.4%)	1 (76.9%)	11 (84.6%)	2 (15.4%)	0 (0.0%)
pT3 N0	12 (19.7%)	32-81	60.5	10 (83.3%)	4.2	12 (100.0%)	2 (16.7%)	5 (41.7%)	0 (0.0%)	7 (58.3%)	4 (33.3%)	1 (8.3%)
pT3 N1a	2 (3.3%)	70-73	71.5	2 (100.0%)	7.3	1 (50.0%)	1 (50.0%)	2 (100.0%)	0 (0.0%)	0 (0.0%)	1 (50.0%)	1 (50.0%)
pT3 N1b	1 (1.6%)	76	76.0	0 (0.0%)	10.5	1 (100.0%)	1 (100.0%)	0 (0.0%)	0 (0.0%)	0 (0.0%)	1 (100.0%)	0 (0.0%)
pT4 N0	2 (3.3%)	61, 75	68.0	1 (50.0%)	6.1	2 (100.0%)	0 (0.0%)	1 (50.0%)	0 (0.0%)	1 (50.0%)	1 (50.0%)	0 (0.0%)
pT4 N1	2 (3.3%)	39, 69	54.0	2 (100.0%)	2.4	2 (100.0%)	1 (50.0%)	1 (50.0%)	0 (0.0%)	0 (0.0%)	1 (50.0%)	1 (50.0%)
Missing stage	18 (29.5%)	39-84	64.4	12 (66.7%)	-	9 (50.0%)	5 (27.8%)	11 (61.1%)	4 (22.2%)	3 (16.7%)	12 (66.7%)	2 (11.1%)
Total	61 (7.52%)	19-84	59.9	48 (78.69%)	4.5	43 (70.49%)	12 (19.67%)	26 (42.62%)	6 (9.83%)	29 (47.54%)	25 (40.98%)	7 (11.47%)

**Medulary carcinoma (N = 54)**												

pT1 N0	2 (3.7%)	37, 52	44.5	2 (100.0%)	0.6	0 (0.0%)	1 (50.0%)	0 (0.0%)	0 (0.0%)	1 (50.0%)	1 (50.0%)	0 (0.0%)
pT1 N1b	5 (9.3%)	16-66	37.4	2 (40.0%)	1.1	2 (40.0%)	2 (40.0%)	3 (60.0%)	4 (80.0%)	1 (20.0%)	3 (60.0%)	1 (20.0%)
pT2 N0	3 (5.6%)	47, 56, 66	56.3	3 (100.0%)	3.4	1 (33.3%)	0 (0.0%)	1 (33.3%)	0 (0.0%)	2 (66.7%)	1 (33.3%)	0 (0.0%)
pT2 N1b	4 (7.4%)	13-61	31.8	4 (100.0%)	3.5	2 (50.0%)	2 (50.0%)	2 (50.0%)	3 (75.0%)	0 (0.0%)	4 (100.0%)	0 (0.0%)
pT3 N1b	10 (18.5%)	8-72	53.8	4 (40.0%)	4.0	5 (50.0%)	0 (0.0%)	8 (80.0%)	10 (100.0%)	0 (0.0%)	9 (90.0%)	1 (10.0%)
pT4a N1b	11 (20.4%)	21-72	42.3	5 (45.5%)	4.5	6 (54.5%)	0 (0.0%)	9 (81.8%)	11 (100.0%)	0 (0.0%)	9 (81.8%)	2 (18.2%)
Missing stage	19 (35.2%)	14-78	41.1	11 (57.9%)	-	11 (57.9%)	6 (31.6%)	14 (73.7%)	12 (63.2%)	0 (0.0%)	16 (84.2%)	3 (15.8%)
Total	54 (6.66%)	8-78	43.9	31 (57.40%)	2.9	27 (50.00%)	11 (20.37%)	37 (68.52%)	40 (68.96%)	4 (7.41%)	43 (79.63%)	7 (11.86%)

**Poorly differentiated carcinoma (N = 11)**												

pT2 N0	1 (9.1%)	72	72.0	1 (100.0%)	2.5	0 (0.0%)	1 (100.0%)	0 (0.0%)	0 (0.0%)	0 (0.0%)	0 (0.0%)	1 (100.0%)
pT2 N1	1 (9.1%)	44	44.0	1 (100.0%)	3.0	1 (100.0%)	1 (100.0%)	1 (100.0%)	1 (100.0%)	0 (0.0%)	0 (0.0%)	1 (100.0%)
pT3 N0	2 (18.2%)	63, 67	65.0	0 (0.0%)	8.4	2 (100.0%)	0 (0.0%)	1 (50.0%)	1 (50.0%)	0 (0.0%)	0 (0.0%)	1 (50.0%)
p T4a N1b	5 (45.5%)	49, 84	60.6	3 (60.0%)	8.7	3 (60.0%)	0 (0.0%)	1 (20.0%)	5 (100.0%)	0 (0.0%)	4 (80.0%)	1 (20.0%)
pT4b	1 (9.1%)	72	72.0	1 (100.0%)	6.0	1 (100.0%)	0 (0.0%)	1 (100.0%)	1 (100.0%)	0 (0.0%)	0 (0.0%)	1 (100.0%)
Missing stage	1 (9.1%)	44	44.0	1 (100.0%)	-	1 (100.0%)	1 (100.0%)	1 (100.0%)	1 (100.0%)	0 (0.0%)	1 (100.0%)	0 (0.0%)
Total	11 (1.36%)	44-84	59.6	7 (63.64%)	5.7	8 (72.72%)	3 (27.27%)	5 (45.45%)	9 (81.82%)	1 (9.09%)	5 (45.45%)	4 (36.36%)

**Anaplastic carcinoma (N = 6)**												

IV B	2 (33.3%)	66, 74	70.0	1 (50.0%)	-	0 (0.0%)	0 (0.0%)	0 (0.0%)	2 (100.0%)	0 (0.0%)	0 (0.0%)	2 (100.0%)
IV C	4 (66.7%)	44-72	57.8	1 (25%)	-	3 (75.0%)	-	4 (100.0%)	4 (100.0%)	0 (0.0%)	2 (50.0%)	2 (50.0%)
Total	6 (0.74%)	44-74	63.0	2 (33.33%)	-	3 (50.00%)	0 (0.00%)	66.67%	6 (100.00%)	0.00%	33.33%	66.67%

Multifocal disease was identified in 301 patients (37.1%). Among the PTC cases, 41.8% were multifocal, and for other types multifocal disease was found in 11.5% of follicular carcinomas, and in 18.5% of medullary carcinoma patients. We did not analyze the subtypes of the medullary carcinomas (sporadic, familial or related to syndromes) due the few number of cases.

Thirty-three patients had simultaneously of their TC second primary malignancies. The most common was breast cancer in 10 patients, followed by colon cancer in 9 patients. Other malignancies diagnosed were lymphomas, lung cancer, prostate cancer, endometrial cancer and melanoma.

Of the DTC patients, 247 (33.4%) had nodal metastasis at presentation, and 44.1% were alive and disease free at the last follow-up.

One hundred sixty-five patients were diagnosed with recurrent disease, 74 of whom had distant metastasis. Among the patients with distant metastasis, four were alive and disease free at the last follow-up, 54 were alive with the disease, and 16 had died from the TC. In the group of patients who had recurrent disease but did not have distant metastasis (91 patients), 63 were alive and disease free, 24 were alive with disease, and four were deceased (one from an unrelated cause and the others from the disease).

Distant metastasis was diagnosed in 125 cases, 92 after the initial surgical treatment. The lung was the most frequent location, seen in 56 patients; 22 had lung and bone metastasis; 19 had only bone metastasis; 13 had lung, liver and bone disease; and three had liver and bone metastasis or only liver metastasis. Thirty of these patients were treated with radioiodine, 13 received target therapy or chemotherapy, and four received external radiation.

Anaplastic thyroid cancer was diagnosed in six patients, all with persistent disease. Four died from the TC between one and 14 months, with a mean of 6 months. Two were alive at the end of this study, having follow-ups of 6 and 13 months.

Persistence or recurrence was seen in 242 cases (29.8%). The mean age at diagnosis among these patients was 50.6 years, similar to that of the entire group. One hundred forty-two patients had PTC, 34 had follicular carcinomas, 50 had medullary carcinomas, and 16 had poorly differentiated or anaplastic carcinomas. Some data related to staging were not available, but 11 patients had nodal metastasis at diagnosis.

**Figure 2:**
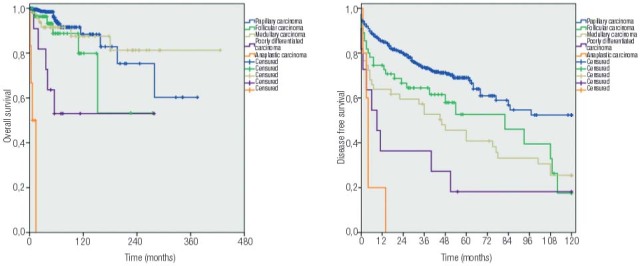

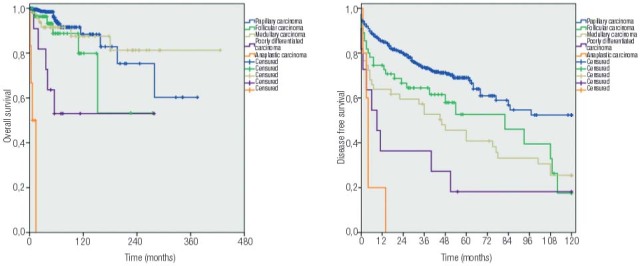
Kaplan-Meier curves demonstrating the overall survival and disease-free survival for each type of thyroid carcinoma. For overall survival the cumulative rate was of 60.3%, 53.2%, 81.3%, 53.0% and 0.0% for, respectively, papillary, follicular, medullary, poorly differentiated and anaplastic carcinoma. Considering disease-free survival, the cumulative rate was 52.4%, 17.6%, 25.5%, 18.2% and 0.0% for, respectively, papillary, follicular, medullary, poorly differentiated and anaplastic carcinoma.

At the last follow-up, 629 (77,6%) patients were alive and disease free, 141 (17.4%) were alive with disease, and 41 were deceased, 37 due to causes related to the TC. Kaplan-Meier curves (Figure 2) identified lower cumulative overall survival for poorly differentiated and anaplastic carcinoma in comparison with other histological types, and also a progressive decrease in disease-free survival in 10-year period for papillary, follicular, medullary, poorly differentiated and anaplastic carcinoma.

## DISCUSSION

We analyzed a considerable sample of patients who had thyroid carcinomas and underwent some type of surgical treatment. However, when we stratified our patients by histological tumor types and stages, some groups become small and insufficient for comparisons with statistical relevance. Although most patients had differentiated carcinomas of the thyroid, we also had cases of medullary carcinomas, poorly differentiated and anaplastic carcinomas, and malignancies with widely varying biological behaviors, which made it challenging to analyze them as a single disease entity. Therefore, we prefer to maintain a purely descriptive analysis to outline an epidemiological profile of these patients.

An analysis of differentiated tumors shows a predominance of female patients, particularly for papillary carcinoma. We have a ratio of 4.4 women for every man affected by differentiated neoplasms, following the proportion observed in the city of São Paulo. The same tendency is observed in cases of anaplastic/poorly differentiated carcinomas, with a ratio of 1.8 women for each affected man. Among patients affected by medullary carcinoma, the ratio of 1.3 women for every man is less than previously reported (4).

In an epidemiological study conducted in São Paulo in 2008, it was found that 2.5% of adults with TC were lean (BMI < 18.5 kg/m^2^), 49.9% had a normal weight (BMI 18.5 to 24.9 kg/m^2^), 34.3% were overweight (BMI 25 to 29.9 kg/m^2^), and 13.2% were obese (BMI greater than or equal to 30 kg/m^2^) (5). Although the average age of our sample was 50.5 years, it was composed of individuals whose diagnoses were made between ages 8 and 87 years, and the mean BMI was not homogeneous among the different age groups in the population, making difficult this analysis. The rate of lean patients was calculated to be 2.3%; 30.1% were eutrophic, and 67.6% were overweight and obese. The growth rates of overweight and obesity in the population has been associated with increased incidence of different cancers, including thyroid (5,6). Parallel curves of growth were observed in these two situations. Preliminary studies, however, could not associate high levels of body mass with medullary carcinoma (6).

Although the survey was conducted in a public hospital that usually treats individuals affected by advanced disease, a considerable percentage of our patients had early-stage disease, classified as pT1 (303 differentiated carcinomas staged as pT1N0, 205 of which were papillary carcinomas). Many carcinomas were characterized as incidentalomas, following the current trend (7,8). However, the outcome was not always favorable in these patients and included associated deaths, as shown in Table 1. Two women diagnosed with papillary carcinoma pT1bN0 at ages 61 and 65 years died, one due to distant metastasis diagnosed 23 months after the initial diagnosis and the other due to the local recurrence diagnosed 84 months after the thyroidectomy. Both had focal capsular invasion and underwent radioiodine therapy and external radiation therapy as complementary treatments.

Four women and one man aged 20 to 56 years were alive and with disease after receiving treatment for papillary thyroid carcinomas pT1N0. Three had distant metastases (one with distant metastasis and local disease and one with unresectable disease). The follow-up periods ranged from 39 to 105 months (66 months on average), and recurrences were diagnosed an average of 30 months after primary treatment.

Among all patients who had papillary carcinoma and were staged as pT1, the recurrence rate was estimated at approximately 6.5%, similar to that reported by other authors (9). We observed, in turn, a cure rate greater than 99% in patients with papillary microcarcinomas (≤ 1 cm in diameter), with no evidence of regional disease at diagnosis, as reported in the literature (10). However, papillary carcinomas staged from pT3N1a (112 patients) were associated with deaths in 8.9% of patients (10 deaths), and 26.8% (30) of the individuals were alive and with disease at the end of follow-up. The average age of these patients was 56.7 years at diagnosis, higher than the average age of 45.4 years among those staged as pT1N0/N1, pT2N0/N1, pT3N0. Associations with older age (1,11), multifocality (7,10), and the presence of lymph node metastases (7,12) with advanced stages and prognosis were reported in differentiated thyroid carcinoma. Of the 68 patients who had differentiated carcinoma and developed distant metastases (9.2%), 46 were age 50 years or older at the diagnosis of their primary tumors, three of whom were less than 20 years old (mean 55.2 years).

The patients with follicular carcinoma followed the trend described in the literature (12) regarding an older age at diagnosis, compared to papillary carcinoma. However, approximately 52% of these patients had cancers at stage pT2N0 or upper, as those who had papillary carcinomas, contradicting reported findings that associated initial diagnosis with advanced stage (12).

Among the 35 patients who could be properly staged, those with medullary carcinoma had, at diagnosis, an 85% rate of cervical lymph node metastases. Studies reported rates from 50-80% (13). Among patients with primary tumors staged as pT1, this rate was estimated at 64.3%. No patient in this group was submitted to prophylactic thyroidectomy because of familial disease. All had thyroid cancer diagnosed by imaging studies. The persistence rates were higher than recurrences diagnosed 6 months after normalization of serum calcitonin titles, and only three patients who had cervical lymph node metastases diagnosed during surgery evolved without biochemical evidence of disease (positive rates of serum calcitonin) at the end of follow-up, which was also expected (14).

Most cases of poorly differentiated thyroid cancer treated at ICESP are not eligible for surgical treatment at the time of their presentation. This series included only the ones that were submitted to some kind of surgical treatment and therefore cannot represent all poorly differentiated thyroid cancer. So we could not attempt to establish prognostic and outcome data. Amongst the eleven poorly differentiated cases, we had a persistence of disease after surgery above 80%, and mortality of 35%. Of the four patients that died from the disease, three had distant metastasis. Reports on this specific topic are not very frequent and there is some inconsistency in the inclusion of anaplastic carcinoma in the same analysis of poorly differentiated, making it hard to establish really reliable data (15).

In the six patients with anaplastic carcinomas, we observed the highest average age at diagnosis by histologic type (63 years) and the highest mortality rates (66.7%), as also observed by other authors (16,17). It is important to note that the two patients still alive were followed up with at 6 and 13 months and will likely evolve with the same outcome. The aggressiveness of this cancer, often associated with poor prognosis surgical and non-resectability, is well known (16,17). Three patients in this group also had some form of non-surgical cancer treatment but without success. The poorly differentiated carcinoma is considered an intermediate step between differentiated carcinomas and anaplastic, and despite its aggressiveness, therapeutic control rates are better as also observed (17).

Excluding patients who received their diagnoses previously at other hospitals, our patients were followed for an insufficient period of time for prognostic comparison, however the Kaplan-Meier curves demonstrated visually this fact. The final status “alive without disease” should be interpreted with caution due to the relatively short follow-up time. However, our study documented well the cases with distant metastases, recurrence and active disease.

The proportions observed regarding the histological types are compatible with those described for thyroid carcinoma (16,18,19). We reported 91% of differentiated tumors (83.7% of papillary carcinomas) in contrast with the rate of 72% reported in an epidemiological study of patients from São Paulo (18). However, our sample should not be considered a representation of the population.

In a descriptive study, we traced a broad profile of individuals admitted to our service for surgical treatments for TC. The observed endpoints follow known standards of prognosis for each histological type analyzed. Our plans include continuous updates.
